# Effects of daily probiotic supplementation with *Lactobacillus acidophilus* on calcium status, bone metabolism biomarkers, and bone mineral density in postmenopausal women: a controlled and randomized clinical study

**DOI:** 10.3389/fnut.2024.1401920

**Published:** 2024-07-01

**Authors:** Iskandar Azmy Harahap, Małgorzata Moszak, Magdalena Czlapka-Matyasik, Katarzyna Skrypnik, Paweł Bogdański, Joanna Suliburska

**Affiliations:** ^1^Department of Human Nutrition and Dietetics, Faculty of Food Science and Nutrition, Poznań University of Life Sciences, Poznań, Poland; ^2^Department of Treatment of Obesity, Metabolic Disorders and Clinical Dietetics, Poznań University of Medical Sciences, Poznań, Poland

**Keywords:** probiotic, calcium status, bone health, DXA (dual-energy X-ray absorptiometry), menopause

## Abstract

**Background:**

Menopause poses significant health risks for women, particularly an increased vulnerability to fractures associated with osteoporosis. Dietary interventions have emerged as promising strategies, focusing on mitigating the risk of osteoporosis rather than solely addressing the established disease. This 12-week randomized controlled trial aimed to analyze the effects of consuming *Lactobacillus acidophilus* probiotics on calcium levels, biomarkers of bone metabolism, and bone mineral density (BMD) profiles in postmenopausal women.

**Methods:**

Fifty-five participants were randomly assigned to receive either a placebo (*n* = 25) or the probiotic *L. acidophilus* UALa-01™ (*n* = 30) daily via oral intervention. Throughout the study, evaluations included body composition, blood biochemical parameters, serum calcium levels, and biomarkers of bone metabolism. Additionally, Dual-energy X-ray absorptiometry was used to measure BMD profiles.

**Results:**

The findings delineated that the probiotic group experienced a decrease in serum calcium levels compared to their initial levels. However, hair calcium levels and biomarkers related to bone metabolism showed no notable changes within this group. Consumption of probiotic *L. acidophilus* also seemed to prevent fluctuations in bone turnover markers. Moreover, there were no significant alterations in BMD levels at the lumbar spine, left femur, and total body in the probiotic group. Additionally, probiotic intake led to favorable outcomes by significantly reducing both body fat and visceral fat during the intervention period. Conversely, an adverse effect of consuming probiotic *L. acidophilus* was observed with a significant increase in glucose concentration.

**Conclusion:**

In conclusion, the consumption of *L. acidophilus* probiotics daily for 12 weeks among postmenopausal women does not affect the profile of BMD, but it may help in stabilizing bone turnover. It is important to note that most measured parameters were within the normal range for this population. However, it is worth noting that 3 months of probiotic supplementation could potentially disrupt calcium and glucose status in postmenopausal women.

## 1 Introduction

Menopause, a natural biological transition, marks the end of a woman’s reproductive years and is typically characterized by hormonal changes, particularly a decline in estrogen levels. This phase, occurring between the ages of 40 and 60 years, signifies a crucial period in a woman’s life, carrying significant implications for her health and overall wellbeing. Women experiencing menopause face various health challenges due to hormonal fluctuations and metabolic changes, making them particularly vulnerable (1). Among these challenges, osteoporosis emerges as a major concern. Osteoporosis is a skeletal condition marked by reduced bone mineral density (BMD) and increased susceptibility to fractures, posing substantial health risks and complications for postmenopausal women (2, 3). According to WHO guidelines, the primary sites for measuring BMD are the proximal femur and the lumbar spine. Typically, BMD assessments are conducted on the L1–L4 section of the lumbar spine (4). Although both femurs can be assessed, the left femur is commonly chosen for diagnostic purposes as per standard practice (5).

Apart from osteoporosis, menopause is linked to a range of metabolic risks, including cardiovascular disease, obesity, and metabolic syndrome. These risks stem from changes in hormonal balance and metabolic function during menopause (6). Fragility fractures, particularly those affecting the spine and hip, contribute significantly to greater morbidity and mortality. The costs associated with fractures increased from €29.6 billion in 2010 to €37.5 billion in 2017, with fragility fractures causing 2.6 million disability-adjusted life years in European countries in 2016 (7). Moreover, menopause profoundly affects calcium status, a vital mineral crucial for bone health and overall metabolic function (8). Calcium is essential for bone formation, muscle contraction, nerve transmission, and blood clotting, playing a pivotal role in maintaining optimal health (9, 10). However, disruptions in calcium metabolism often occur during menopause, leading to imbalances in calcium levels and potentially increasing the risk of osteoporosis and other metabolic disorders (11). Understanding the interaction between menopause, calcium status, and metabolic health is crucial for developing effective strategies to mitigate the adverse health outcomes associated with menopause.

Treating menopausal symptoms presents significant challenges, particularly due to the limitations and adverse effects of hormone therapy. Although hormone therapy has traditionally been crucial for managing menopausal symptoms and reducing the risk of osteoporosis-related fractures, its use comes with notable adverse effects and safety concerns. One notable concern is the increased risk of cancer associated with prolonged hormone therapy, particularly breast cancer and endometrial cancer. These risks have led to hesitancy among healthcare providers and patients in considering hormone therapy as a long-term solution for menopausal osteoporosis (12). Moreover, existing pharmacological treatments for osteoporosis, such as bisphosphonates and SERMs (selective estrogen receptor modulators), have limitations that hinder their sustained use. While bisphosphonates effectively lower fracture risk, they are associated with gastrointestinal side effects such as esophageal irritation and ulceration, as well as rare but severe adverse events like osteonecrosis of the jaw and atypical femoral fractures (13). Similarly, although SERMs help maintain bone density and reduce fracture risk, they are associated with an increased risk of venous thromboembolism and hot flashes (14). Given the limitations and safety concerns associated with existing treatments, there is an urgent need to explore alternative therapeutic approaches that offer both effectiveness and long-term safety for menopausal women. Consequently, there is growing interest in identifying new preventive and treatment strategies that can ensure safe and effective long-term management of osteoporosis in menopausal women.

During menopause, alterations in the composition of gut microbiota, termed dysbiosis, have been observed, potentially impacting various physiological processes, including bone health (15). This phenomenon underscores the significance of the gut-bone axis, a two-way communication pathway between gut microbiota and the skeletal system (16).

Probiotics, recognized for their ability to modulate gut microbiota composition and function, have garnered attention for their potential role in regulating calcium homeostasis and bone metabolism. Specifically, probiotics have been shown to influence calcium absorption in the intestine, thereby affecting overall calcium status and bone mineralization (17). Moreover, probiotics have been implicated in regulating key biomarkers associated with bone turnover, such as C-terminal telopeptide of type I collagen (CTX) and tartrate-resistant acid phosphatase isoform-5b (TRAP-5b), which indicate bone resorption rates (18). In addition to bone resorption markers, probiotics have also been found to impact biomarkers indicative of bone formation, including bone-specific alkaline phosphatase (BSAP) and N-terminal propeptide of type I procollagen (PINP) (19). Probiotics offer a promising approach to enhance bone health, with emerging evidence suggesting their potential to reduce risks associated with osteoporosis (20). Numerous studies have demonstrated the beneficial effects of probiotics on bone health, ranging from cellular assays to animal models and clinical trials involving menopausal conditions (21–23). Among the various probiotic strains studied, *Lactobacillus acidophilus* emerges as a particularly promising candidate. This specific strain has garnered significant attention due to its proven effectiveness in promoting bone health and preventing the progression of osteoporosis (24). *L. acidophilus* may aid in the process of osteogenic differentiation during bone mineralization, as evidenced by studies conducted on human osteosarcoma Saos-2 cells (25). In our previous research, we extensively investigated the effects of *L. acidophilus* supplementation using *in vitro* digestion models and healthy female rats (26–30), shedding light on its potential implications for calcium status and bone health.

The decision to conduct a human trial with *L. acidophilus* supplementation was rooted in several considerations. While our previous studies involving healthy rats did not yield statistically significant effects on calcium transport and bone metabolism biomarkers (27), our study involving healthy rats revealed noteworthy trends, such as a significant increase in calcium content in the femur of female rats following *L. acidophilus* DSM079 supplementation (31). It revealed promising trends suggesting a potential impact of *L. acidophilus* on calcium metabolism and bone health. Additionally, our *in vitro* cell study provided valuable insights by demonstrating that *L. acidophilus* may enhance osteogenic differentiation in Saos-2 cells (25), indicating its potential mechanisms of action on bone health. These findings underscored the need for further exploration in a human study. Despite the absence of a preclinical study specifically on a model of postmenopausal osteoporosis, existing literature has documented significant effects of *L. acidophilus* on bones and calcium metabolism in other experimental contexts (20). Therefore, based on these collective findings of *L. acidophilus* intake, we deemed it pertinent to investigate its potential benefits in postmenopausal women.

However, despite promising findings from both preclinical and clinical studies, there are still gaps in our understanding of how probiotics precisely influence the gut-bone axis and bone health. While dual-energy X-ray absorptiometry (DXA) serves as the gold standard for evaluating BMD (32), there remains a notable research gap in understanding the potential impact of probiotic supplementation on bone health in postmenopausal women. Despite extensive research on the effectiveness of probiotics in various health conditions, including gastrointestinal disorders (32) and immune function (33), few studies have explored their effects on bone metabolism and BMD profiles in this population. This dearth of research hinders our ability to fully comprehend the therapeutic potential of probiotics as a novel intervention for managing osteoporosis in postmenopausal women. Therefore, the present study aimed to investigate the effects of 12 weeks of oral and daily consumption of probiotic *L. acidophilus* UALa-01™ on selected parameters of calcium status, bone metabolism biomarkers, and BMD profiles in postmenopausal women. It is crucial to emphasize that our hypothesis suggests a positive impact of probiotic supplementation with *L. acidophilus* on calcium status, bone metabolism biomarkers, and BMD profiles in postmenopausal women. This investigation seeks to address a notable research gap by shedding light on the potential benefits of probiotics in improving calcium status and bone health, as well as potentially mitigating the risk of osteoporotic fractures among this demographic. By exploring this area, we aim to provide novel insights that could have significant implications for public health strategies targeting postmenopausal women.

## 2 Materials and methods

### 2.1 Ethics of human clinical study

The clinical trial protocol for this study was approved by the Ethics Committee of Poznań University of Medical Sciences, Poland (approval no. 668/21 issued on 23 September 2021). Furthermore, the trial is registered with ClinicalTrials.gov under the identifier NCT05332626.^1^ This investigation rigorously adheres to a comprehensive set of ethical and regulatory standards, as outlined in various legislations and regulations. Additionally, it complies with the CONSORT guidelines, ensuring transparent reporting of the study methodology and results. The study also aligns with the Declaration of Helsinki of the World Medical Association, which incorporates ethical principles for medical research involving humans and adheres to the guidelines set forth by the International Conference on Harmonization of Good Clinical Practice. This meticulous adherence to ethical and regulatory frameworks ensures the protection of participants’ rights, safety, and wellbeing throughout the entirety of the clinical trial.

### 2.2 Study design: enrolment, allocation, and randomization

Initial assessments of postmenopausal patients were conducted at the Department of Treatment of Obesity, Metabolic Disorders, and Clinical Dietetics, Poznań University of Medical Sciences, Poland. To qualify for participation, individuals had to meet specific inclusion criteria, including providing written informed consent, being aged between 45 and 70 years, with a body mass index (BMI) between 27.0 and 34.9 kg/m^2^, and confirming postmenopausal status by experiencing spontaneous amenorrhea for 12 months or longer. Exclusion criteria included a diagnosis of diabetes, secondary obesity, gastrointestinal diseases, recent use of dietary supplements, or pharmacotherapy for lipid disorders or hypertension within the 3 months before enrollment, presence of active clinically significant inflammatory processes, recent antibiotic intake within the month before enrollment, participation in a body mass management study, or use of drugs known to affect body mass. Additionally, individuals who were current smokers or exhibited abuse of alcohol or drugs were also excluded from participation. Those meeting any exclusion criteria were not included in the study and were required to withdraw immediately if any exclusion criteria were identified during the course of the study. The study enrolled 64 subjects who met all inclusion criteria and provided written informed consent, undergoing the randomization procedure.

**FIGURE 1:**
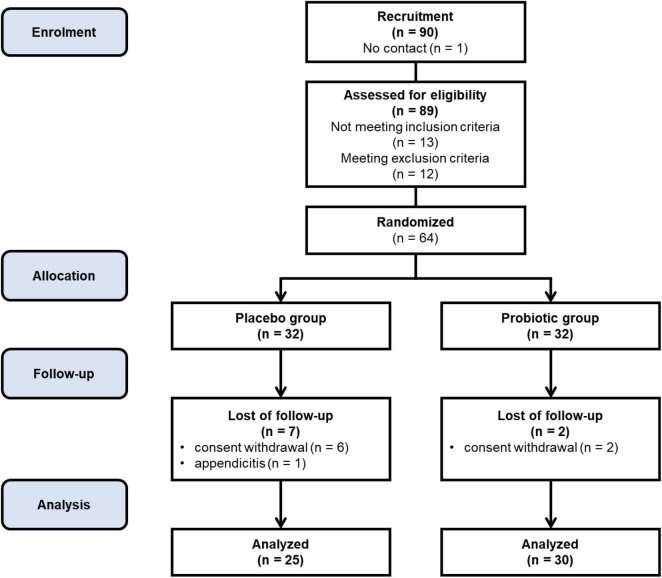
Flowchart of enrollment, allocation, and randomization of participants.

The study included 64 participants who were randomly assigned to two groups: the placebo group (*n* = 32) and the probiotic group (*n* = 32). Patient allocation was conducted in a blinded manner, ensuring both subjects and investigators remained unaware of the distribution. To uphold confidentiality, each participant received a unique identifier code from the study personnel. The randomization process was computer-generated, preventing any adjustments by the researcher directly involved with the patients. Despite stringent randomization procedures, baseline discrepancies were observed between the probiotic and placebo groups, particularly in body composition parameters and serum calcium levels. These differences may be attributed to the inherent challenges in achieving perfect homogeneity in participant characteristics, given the specific inclusion and exclusion criteria applied. While efforts were made to minimize these variations, such as rigorous screening protocols, it is acknowledged that residual confounding factors may have influenced the study outcomes. Out of the initial participants, 55 women completed the intervention and underwent subsequent statistical analysis, with 25 from the placebo group and 30 from the probiotic group. Figure 1 illustrates the study’s progression through a flowchart, showing the number of patients lost to follow-up and explaining the reasons for their exclusion. Moreover, the analyses involved daily records, body composition assessment using the InBody device, serum bone metabolism biomarkers using ELISA, hair and serum calcium analysis using AAS, and bone density profiles using the DXA scanner. The study intervention and measurements were conducted between January 2022 and December 2023.

**FIGURE 2:**
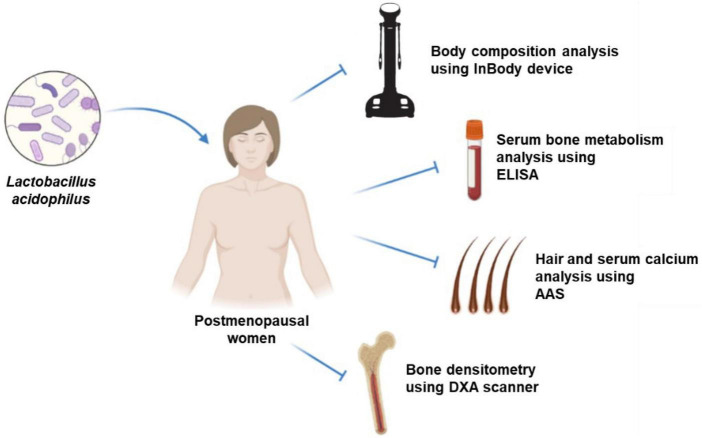
Experimental design of intervention and assessment; ELISA, enzyme-linked immunosorbent assay; AAS, atomic absorption spectrometry; DXA, dual-energy X-ray absorptiometry.

The probiotic groups were given a daily oral dose of 1 × 10^9^ colony-forming units of *L. acidophilus* UALa-01™, with the excipient containing microcrystalline cellulose, silica, magnesium stearate, and a gelatin natural capsule. The probiotics were administered after meals in the morning time at 08:00 a.m (± 1 h). In contrast, the placebo group received only the excipient orally, designed to be indistinguishable in taste, smell, and appearance from the probiotic mixture. The placebo also consisted of microcrystalline cellulose, silica, magnesium stearate, and a gelatin natural capsule. The study intervention lasted for 12 weeks, during which participants were explicitly instructed not to modify their regular physical activity. Figure 2, a research flowchart, visually outlines the research process and provides an overview of the assessment of collected samples.

### 2.3 Assessment of nutritional values of daily diet

The nutritional content of participants’ daily diets was assessed using a standardized methodology involving a 3-day food recall procedure. This dietary assessment method utilized the 6.0 Diet Program (National Food and Nutrition Institute, Warsaw, Poland), known for its precision and reliability in capturing dietary intake data. Participants were given a questionnaire to record their dietary intake for 3 days before the start of the intervention and for 3 days preceding its conclusion. The 3-day food recall method allowed for the collection of detailed information on participants’ dietary intake, including the types and quantities of foods consumed, meal timing, and cooking methods. Trained research personnel supervised the dietary recalls to ensure accuracy and consistency in data collection. Participants were guided through recalling their dietary intake for the specified periods, with a focus on recording all foods and beverages consumed, along with portion sizes and preparation methods. After completing the dietary recall questionnaires, the collected data were entered into the 6.0 Diet Program for analysis. This software facilitated the calculation of nutrient intakes based on reported food consumption, enabling the assessment of energy, macronutrient, and micronutrient intake levels. Specifically, nutrient compositions such as energy (Kcal), protein (gram), fat (gram), carbohydrates (gram), fiber (gram), calcium (gram), phosphorus (gram), calcium/phosphorus ratio, and vitamin D (gram) were analyzed. The analysis provided insights into the overall nutritional adequacy of participants’ diets and allowed for comparisons between baseline and intervention phases to evaluate the impact of the dietary intervention on dietary quality and nutrient intake patterns.

### 2.4 Body composition and anthropometric measurements

Baseline and posttrial assessments included anthropometric measurements and body composition analyses. In a dedicated metabolic laboratory, all anthropometric measurements were meticulously conducted with participants wearing light clothing and no shoes, following an overnight fast and a period of rest. Body mass (kg) was measured using electronic scales with precision to the nearest 0.1 kg. BMI was calculated by dividing the mass by the square of the height (kg/m^2^). Waist circumference (cm) was measured to the nearest 0.5 cm at the end of normal expiration between the iliac crest and the lower rib, providing additional anthropometric insights. Hip circumference (cm) was measured at the widest part of the buttocks, also to the nearest 0.5 cm. The waist-hip ratio was calculated by dividing the waist circumference by the hip circumference. Body composition assessment utilized the InBody 270 system (Cerritos, CA, USA), ensuring a comprehensive analysis of body composition parameters. The InBody 270 device employs a multifrequency 8-point tetrapolar touch electrode system to accurately measure body composition. By applying a range of frequencies to assess impedance across various body segments, the system allows for precise analysis of body adiposity index (BAI) (%), minerals (kg), and lean body mass (kg). Measurements were taken by placing electrodes on the hands and feet, with the device applying a low-level electrical current to measure impedance. Proprietary algorithms were used to analyze impedance data and generate body composition results within seconds. To ensure accuracy and reliability, participants stood barefoot on the device’s foot electrodes while holding onto the hand electrodes during measurements. The InBody 270 then generated a comprehensive body composition report, including values for skeletal muscle mass (kg), body fat percentage (%), and visceral fat level (point). Quality control measures were implemented to maintain consistency and precision throughout the study period. In addition, we acknowledge that certain factors related to participants’ routines or health conditions could potentially affect the accuracy of the results. These factors may include hydration status, meal timing, exercise regimen, and specific health conditions affecting body composition (34). The InBody 270 was equipped with user-friendly software for easy data management and interpretation. Participant data were securely stored within the device’s database, and results were automatically generated and printed for review by the research team. Before data collection, all study personnel underwent training on the proper use of the InBody 270 to minimize measurement error and ensure standardized procedures across all participants.

### 2.5 Serum and hair sample collection

Blood samples were obtained from a forearm vein after an overnight fast, both at baseline and upon completion of the trial. These samples were collected into serum-separating tubes for subsequent analysis of biochemical markers and minerals. Whole-blood morphological and biochemical parameters at baseline and endline were measured in a certified commercial laboratory (Alab Laboratories, Poznań, Poland) immediately after collection. The serum samples were promptly stored at −80°C to maintain their integrity until analysis. Concurrently, hair samples were gathered at the commencement and conclusion of the trial. Specifically, a hair sample was obtained from the occipital region, and when weighed as a whole, the average weight of the sample was found to be 0.5 grams. The samples were securely stored in individually labeled paper bags. Dyed and permed hair was not collected. Patients were explicitly instructed on the importance of adhering to this collection procedure for obtaining reliable results. Throughout the study, participants were instructed not to use hair spray or hair dye to ensure the purity of the collected samples. Additionally, the mass of each hair sample was meticulously measured. Hair samples were washed in acetone and deionized water, and then dried at 105°C.

### 2.6 Calcium content measurement

Calcium contents in serum and hair samples were analyzed using flame atomic absorption spectrometry (AAS-3, Carl Zeiss, Jena, Germany) following appropriate dilution with deionized water and the addition of 0.5% Lanthanum (III) chloride (Merck KGaA, Darmstadt, Germany). The quantification of calcium content was carried out at a specific wavelength of 422.7 nm. To assess the precision and reliability of this analytical method, certified reference materials, including human serum (Hum Asy Control 2, Sero, Billingstad, Norway) and human hair (NCS DC73347a LGC, Teddington, UK), were used. Results obtained from analyzing these reference materials demonstrated a notably high level of method accuracy, achieving a calculated accuracy rate of 91–93% for calcium quantification.

### 2.7 Bone biomarkers measurement

Commercial enzyme-linked immunosorbent assay (ELISA) kits procured from Qayee Bio-Technology Co., Ltd., Shanghai, China, were used along with absorption spectrophotometry (LEDetect96, Labexim, Lengau, Austria) to measure serum levels of markers associated with bone metabolism. Specifically, CTX and TRAP-5b, indicating bone resorption, were assessed, along with BSAP and PINP, biomarkers reflecting bone formation.

### 2.8 DXA bone mineral density assessment

All participants, both before and after the intervention, underwent a DXA scan at the Department of Human Nutrition and Dietetics, Poznań University of Life Sciences, Poland, administered by the same researcher using a GE Lunar Prodigy^®^ machine (General Electric Healthcare, Madison, WI, USA). During the procedure, participants were asked to remove all metal components from their clothing and accessories to ensure accurate measurements. The BMD (g/cm^2^) of the L1–L4 lumbar spines, left femur, and total body was assessed using the DXA software (enCORE by General Electric Healthcare, Madison, WI, US). Daily rigorous calibration and quality control of the DXA equipment were carried out to maintain the stability and reliability of the system. This meticulous approach to DXA scanning enhances the precision and credibility of the BMD assessments in our study.

### 2.9 Statistical analysis

IBM^®^ SPSS^®^ Statistics version 22 for Windows (Chicago, IL, US) was used for statistical analysis and figure generation. Measurements were presented as mean and median values, accompanied by their corresponding standard deviations with interquartile range. This approach provides a comprehensive depiction of the data distribution and variability, ensuring a thorough understanding of the central tendency and spread within the dataset. In addition to the analysis conducted, the normality of the data distribution was assessed using the Shapiro–Wilk test to ensure adherence to statistical assumptions crucial for subsequent analyses. Statistical analysis was performed to ascertain the significance of observed differences. For comparisons within the same group before and after the intervention (dependent groups), the Wilcoxon matched pairs test was used. For comparisons between the placebo and probiotic groups (independent groups), the Mann-Whitney U test was employed. The predetermined significance threshold for all observed differences was set at a 5% probability level. To investigate the relationships among serum calcium levels, bone biomarkers, and BMD profiles, Spearman’s correlation analysis was employed. Furthermore, careful consideration was given to determining an appropriate sample size, with a minimum requirement of 25 subjects in each group established. This study followed the methodology outlined in the study by Soleimani et al. (35). A power calculation was performed considering a type I error (α) of 0.05 and a type II error (β) of 0.20, resulting in a power of 80%. Based on these parameters, it was determined that a minimum of 25 subjects in each group would be necessary to detect statistically significant differences. This calculation accounted for the primary outcome measures, including changes in serum and hair calcium levels, as well as BMD profiles. This meticulous approach to assessing data distribution and determining sample size contributes to the methodological rigor of the study, enhancing its ability to produce robust and reliable findings within the specified statistical framework.

## 3 Results

### 3.1 Result of nutritional values of food recall

Table 1 depicts the dietary patterns of the study participants at baseline and endline. Before the intervention, no significant differences were observed in dietary intake variables between the placebo and probiotic groups. However, after the 12-week intervention, the probiotic group displayed a noticeable increase in energy, protein, carbohydrates, calcium, and phosphorus intake compared to the placebo group. Moreover, the placebo group exhibited a significant decrease in energy intake from preintervention to postintervention. Conversely, the probiotic group demonstrated a significant increase in dietary calcium intake at endline across all observed variables compared to the baseline period.

**TABLE 1:** Characteristics of dietary pattern from participating subjects at baseline and endline.

Variable	Group	Baseline		Endline		*P*-value	Sig.
		Mean ± SD	Median (IQR)	Mean ± SD	Median (IQR)		
Energy (Kcal)	Placebo	1,792.96 ± 502.88	1,776.50 (1,545.60–2,135.45)	1,576.95 ± 373.62	1,573.20 (1,312.45–1,799.35)	0.01	Sig.
	Probiotic	1,827.56 ± 577.35	1,883.70 (1,442.55–2,262.55)	1,922.93 ± 650.91	1,831.80 (1,423.50–2,414.75)	0.47	NS
	*P*-value	0.97		0.00			
	Sig.	NS		Sig.			
Protein (gram)	Placebo	77.20 ± 27.46	77.90 (66.00 –90.80)	73.62 ± 19.98	73.70 (61.60–82.15)	0.42	NS
	Probiotic	77.86 ± 22.18	80.70 (60.40–92.40)	87.50 ± 28.18	87.90 (62.45–107.55)	0.05	NS
	*P*-value	0.83		0.01			
	Sig.	NS		Sig.			
Fat (gram)	Placebo	70.90 ± 29.80	64.80 (51.55–94.10)	58.08 ± 22.86	54.40 (39.55–74.30)	0.05	NS
	Probiotic	68.52 ± 29.79	65.70 (44.85–82.10)	73.34 ± 32.78	72.70 (50.00–91.05)	0.73	NS
	*P*-value	0.51		0.06			
	Sig.	NS		NS			
Carbohydrates (gram)	Placebo	195.16 ± 63.36	196.60 (152.40–238.60)	177.04 ± 48.98	169.50 (141.15–209.40)	0.12	NS
	Probiotic	205.21 ± 74.61	204.20 (148.30–259.10)	210.04 ± 80.63	190.30 (147.45–280.60)	0.56	NS
	*P*-value	0.87		0.03			
	Sig.	NS		Sig.			
Fiber (gram)	Placebo	24.12 ± 7.91	25.10 (17.05–29.55)	24.34 ± 8.77	24.50 (17.60–28.75)	0.87	NS
	Probiotic	26.37 ± 10.53	26.10 (20.10–32.25)	27.09 ± 10.77	26.90 (18.75–33.60)	0.84	NS
	*P*-value	0.70		0.22			
	Sig.	NS		NS			
Calcium (gram)	Placebo	927.06 ± 354.63	903.50 (659.20–1,177.30)	894.72 ± 381.84	795.70 (627.65–1,068.60)	0.53	NS
	Probiotic	895.82 ± 357.92	816.40 (661.10–1,090.45)	1,115.81 ± 529.76	947.50 (716.30–1,426.00)	0.03	Sig.
	*P*-value	0.38		0.03			
	Sig.	NS		Sig.			
Phosphorus (gram)	Placebo	1,354.52 ± 428.68	1,409.70 (1,114.65–1,596.55)	1,314.72 ± 374.96	1,260.60 (1,050.20–1,472.35)	0.58	NS
	Probiotic	1,467.68 ± 776.82	1,391.50 (1,038.75–1,630.90)	1,563.40 ± 566.34	1,534.00 (1,179.30–1,963.45)	0.12	NS
	*P*-value	0.91		0.01			
	Sig.	NS		Sig.			
Calcium/phosphorus (gram)	Placebo	0.69 ± 0.17	0.71 (0.54–0.81)	0.67 ± 0.17	0.70 (0.57–0.81)	0.37	NS
	Probiotic	0.64 ± 0.18	0.66 (0.52–0.79)	0.72 ± 0.23	0.67 (0.54–0.87)	0.18	NS
	*P*-value	0.22		0.50			
	Sig.	NS		NS			
Vitamin D (gram)	Placebo	3.77 ± 9.31	1.70 (0.75–3.65)	5.17 ± 8.26	1.80 (1.05–4.90)	0.25	NS
	Probiotic	2.41 ± 3.10	2.00 (1.00–2.70)	2.80 ± 2.99	1.80 (0.90–3.15)	0.46	NS
	*P*-value	0.51		0.25			
	Sig.	NS		NS			

SD, standard deviation; IQR, interquartile range. Statistical analysis was conducted using a Wilcoxon two-related samples test with a significance threshold set at *p* = 0.05. Sig., Significance; NS, not significance.

### 3.2 Result of body composition and anthropometric profiles

Table 2 displays the body composition and anthropometric profiles of study participants at baseline and endline. Before the intervention, significant differences were observed between the placebo and probiotic groups, including body mass, BMI, waist circumference, hip circumference, and BAI, with the probiotic group demonstrating lower values in these variables compared to the placebo group. Following the 12-week intervention, the probiotic group exhibited a noteworthy reduction in the percentage of body fat and visceral fat between preintervention and postintervention assessments. However, it is important to note that these changes were not significantly different when compared to the placebo group.

**TABLE 2:** Characteristics of body composition and anthropometric profiles from participating subjects at baseline and endline.

Variable	Group	Baseline		Endline		*P*-value	Sig.
		Mean ± SD	Median (IQR)	Mean ± SD	Median (IQR)		
Body mass (kg)	Placebo	78.30 ± 14.63	75.50 (66.50–88.80)	73.88 ± 13.45	68.90 (65.60–83.30)	0.14	NS
	Probiotic	68.56 ± 13.43	65.30 (58.80–71.70)	72.22 ± 15.21	69.10 (59.60–81.40)	0.40	NS
	*P*-value	0.01		0.65			
	Sig.	Sig.		NS			
Body mass index (kg/m^2^)	Placebo	28.58 ± 4.35	28.10 (24.70–30.90)	27.66 ± 4.41	27.60 (23.90–30.50)	0.35	NS
	Probiotic	25.29 ± 4.76	23.80 (21.90–28.60)	26.35 ± 5.00	24.00 (22.30–29.50)	0.57	NS
	*P*-value	0.01		0.46			
	Sig.	Sig.		NS			
Waist circumference (cm)	Placebo	96.47 ± 12.40	98.00 (86.50–105.00)	93.72 ± 12.04	93.00 (84.50–104.00)	0.39	NS
	Probiotic	86.24 ± 12.13	85.75 (76.50–90.00)	88.04 ± 13.34	89.25 (77.25–96.00)	0.62	NS
	*P*-value	0.01		0.28			
	Sig.	Sig.		NS			
Hip circumference (cm)	Placebo	110.78 ± 8.76	110.00 (103.75–116.50)	107.57 ± 10.51	106.00 (100.75–111.50)	0.15	NS
	Probiotic	102.48 ± 9.54	99.50 (95.00–107.75)	104.19 ± 10.71	103.00 (95.50–111.50)	0.57	NS
	*P*-value	0.01		0.20			
	Sig.	Sig.		NS			
Waist-Hip ratio	Placebo	0.87 ± 0.07	0.89 (0.84–0.91)	0.87 ± 0.09	0.87 (0.82–0.90)	0.48	NS
	Probiotic	0.84 ± 0.07	0.82 (0.79–0.90)	0.84 ± 0.07	0.84 (0.78–0.89)	0.99	NS
	*P*-value	0.14		0.25			
	Sig.	NS		NS			
Body adiposity index (%)	Placebo	34.29 ± 4.16	34.14 (31.50–37.34)	32.86 ± 5.79	33.07 (28.61–36.52)	0.14	NS
	Probiotic	30.91 ± 4.84	28.94 (27.12–35.89)	31.77 ± 5.96	30.48 (27.11–35.73)	0.60	NS
	*P*-value	0.02		0.41			
	Sig.	Sig.		NS			
Minerals (kg)	Placebo	3.15 ± 0.47	3.22 (2.90–3.48)	3.11 ± 0.45	3.03 (2.78–3.40)	0.82	NS
	Probiotic	3.29 ± 0.44	3.26 (3.01–3.53)	3.33 ± 0.42	3.25 (3.01–3.56)	0.23	NS
	*P*-value	0.28		0.06			
	Sig.	NS		NS			
Lean body mass (kg)	Placebo	44.58 ± 7.01	43.30 (41.80–48.30)	45.29 ± 5.61	44.10 (41.10–50.00)	0.44	NS
	Probiotic	46.54 ± 6.09	45.70 (42.10–52.50)	46.79 ± 5.98	46.00 (42.20–52.00)	0.47	NS
	*P*-value	0.47		0.48			
	Sig.	NS		NS			
Skeletal muscle mass (kg)	Placebo	26.21 ± 5.95	24.20 (23.00–28.40)	27.19 ± 7.61	24.60 (22.30–29.90)	0.34	NS
	Probiotic	25.55 ± 3.65	24.80 (22.90–29.20)	25.67 ± 3.63	25.30 (22.90–28.90)	0.54	NS
	*P*-value	0.94		0.98			
	Sig.	NS		NS			
Body fat (%)	Placebo	36.96 ± 7.35	37.40 (32.50–42.30)	37.29 ± 6.80	39.90 (31.60–42.10)	0.84	NS
	Probiotic	34.49 ± 8.28	34.60 (27.60–40.80)	33.91 ± 8.12	34.30 (28.30–39.90)	0.04	Sig.
	*P*-value	0.44		0.25			
	Sig.	NS		NS			
Visceral fat (point)	Placebo	12.96 ± 4.63	11.00 (9.00–18.00)	13.20 ± 4.45	12.00 (10.00–17.00)	0.45	NS
	Probiotic	12.00 ± 5.02	11.00 (8.00–16.00)	11.33 ± 5.27	11.00 (7.00–15.00)	0.00	Sig.
	*P*-value	0.48		0.29			
	Sig.	NS		NS			

SD, standard deviation; IQR, Interquartile Range. Statistical analysis was conducted using a Wilcoxon two-related samples test with a significance threshold set at *p* = 0.05. Sig., significance; NS, not significance.

### 3.3 Result of blood morphology and biochemical parameter profiles

Table 3 illustrates the blood morphology profiles of study participants before and after a 12-week intervention. Before the intervention, significant differences were observed between the placebo and probiotic groups in hemoglobin, hematocrit, and triglyceride levels, with the probiotic group exhibiting significantly lower levels of these variables than the placebo group. Following the intervention period, the placebo group demonstrated significant decreases in hemoglobin and hematocrit values compared to the preintervention period. It is noted that the probiotic group exhibited significantly lower levels of hemoglobin, hematocrit, and triglycerides compared to placebo group at the preintervention period. Conversely, the placebo group demonstrated a significant decrease in hemoglobin and hematocrit levels following the intervention period. However, it is important to note that despite these changes, all values remained within the normal physiological range for postmenopausal women, and there were no significant differences observed between the probiotic and placebo groups. In contrast, the probiotic group exhibited a significant increase in glucose concentration from the preintervention to the postintervention period. However, it is noteworthy that no significant difference in glucose concentration was observed between the probiotic group and placebo group at the postintervention period.

**TABLE 3:** Blood morphology and biochemical parameters in subjects at baseline and endline.

Variable	Group	Baseline		Endline		*P*-value	Sig.
		Mean ± SD	Median (IQR)	Mean ± SD	Median (IQR)		
WBC	Placebo	5.66 ± 0.96	5.37 (4.80–6.30)	6.03 ± 0.89	5.80 (5.59–7.00)	0.06	NS
	Probiotic	5.94 ± 1.08	5.18 (5.60–6.88)	5.32 ± 0.90	5.30 (4.65–5.90)	0.05	NS
	*P*-value	0.65		0.19			
	Sig.	NS		NS			
RBC	Placebo	4.59 ± 0.28	4.54 (4.35–4.82)	4.43 ± 0.35	4.49 (4.29–4.58)	0.09	NS
	Probiotic	4.41 ± 0.36	4.41 (4.24–4.59)	4.35 ± 0.29	4.35 (4.17–4.59)	0.74	NS
	*P*-value	0.08		0.68			
	Sig.	NS		NS			
Hemoglobin (g/dL)	Placebo	14.26 ± 0.87	14.30 (13.80–14.70)	13.55 ± 0.99	14.00 (12.90–14.20)	0.01	Sig.
	Probiotic	13.58 ± 0.93	13.80 (13.05–14.30)	13.71 ± 0.83	13.65 (13.38–14.33)	0.82	NS
	*P*-value	0.02		0.91			
	Sig.	Sig.		NS			
Haematocrit (%)	Placebo	41.72 ± 2.49	42.00 (40.20–43.00)	39.87 ± 2.72	41.00 (37.50–41.80)	0.01	Sig.
	Probiotic	38.79 ± 6.25	40.20 (37.40–41.63)	39.00 ± 6.28	39.80 (38.60–41.63)	0.92	NS
	*P*-value	0.02		0.81			
	Sig.	Sig.		NS			
Platelet	Placebo	259.42 ± 61.53	259.00 (225.00–304.00)	265.16 ± 63.17	273.00 (232.00–302.00)	0.75	NS
	Probiotic	268.84 ± 49.33	263.00 (230.00–306.50)	251.56 ± 48.95	251.00 (215.50–277.00)	0.28	NS
	*P*-value	0.72		0.62			
	Sig.	NS		NS			
TC (mg/dl)	Placebo	234.67 ± 44.53	231.00 (206.00–270.00)	214.22 ± 42.72	208.00 (187.00–240.00)	0.06	NS
	Probiotic	224.35 ± 49.42	226.00 (195.50–241.75)	227.58 ± 42.86	226.50 (187.75–261.00)	0.76	NS
	*P*-value	0.32		0.36			
	Sig.	NS		NS			
HDL (mmol/l)	Placebo	1.59 ± 0.40	1.65 (1.28–1.75)	1.57 ± 0.39	1.53 (1.32–1.74)	0.18	NS
	Probiotic	1.67 ± 0.28	1.68 (1.50–1.79)	1.74 ± 0.65	1.66 (1.48–1.95)	0.44	NS
	*P*-value	0.38		0.38			
	Sig.	NS		NS			
Triglycerides (mmol/l)	Placebo	1.31 ± 0.41	1.32 (0.95–1.63)	1.24 ± 0.50	1.13 (0.86–1.45)	0.41	NS
	Probiotic	1.09 ± 0.33	1.17 (0.80–1.32)	1.12 ± 0.38	1.06 (0.83–1.39)	0.79	NS
	*P*-value	0.02		0.24			
	Sig.	Sig.		NS			
LDL (mg/dl)	Placebo	150.44 ± 43.50	146.00 (112.00–193.00)	131.85 ± 38.19	124.00 (104.00–163.00)	0.07	NS
	Probiotic	139.81 ± 43.47	148.00 (108.75–158.50)	145.00 ± 41.27	148.00 (111.00–180.25)	0.70	NS
	*P*-value	0.41		0.21			
	Sig.	NS		NS			
Glucose (mg/dl)	Placebo	90.93 ± 10.31	89.00 (86.00–92.00)	89.04 ± 8.33	87.00 (83.00–95.00)	0.43	NS
	Probiotic	87.50 ± 8.76	86.00 (82.00–91.25)	96.42 ± 15.29	93.50 (89.00–97.25)	0.01	Sig.
	*P*-value	0.15		0.08			
	Sig.	NS		NS			
Insulin (μU/dl)	Placebo	6.63 ± 3.44	5.20 (4.00–9.00)	6.30 ± 3.18	5.40 (3.30–8.80)	0.75	NS
	Probiotic	6.07 ± 3.44	5.30 (3.23–8.40)	7.11 ± 3.89	6.75 (3.95–10.08)	0.52	NS
	*P*-value	0.71		0.47			
	Sig.	NS		NS			
HOMA-IR	Placebo	1.56 ± 1.07	1.14 (0.87–2.02)	1.42 ± 0.79	1.16 (0.73–2.11)	0.85	NS
	Probiotic	1.36 ± 0.86	1.02 (0.78–1.95)	1.81 ± 1.35	1.50 (0.91–2.48)	0.41	NS
	*P*-value	0.84		0.31			
	Sig.	NS		NS			
hs-CRP	Placebo	0.25 ± 0.20	0.18 (0.09–0.41)	0.58 ± 1.39	0.17 (0.10–0.49)	0.54	NS
	Probiotic	0.34 ± 0.48	0.10 (0.07–0.29)	0.17 ± 0.16	0.13 (0.04–0.21)	0.45	NS
	*P*-value	0.45		0.09			
	Sig.	NS		NS			

WBC, white blood cells; RBC, red blood cells; TC, total cholesterol; HDL, high-density lipoproteins; LDL, low-density lipoproteins; HOMA-IR, Homeostatic Model Assessment for Insulin Resistance; hs-CRP, high-sensitivity C-reactive Protein. SD, standard deviation; IQR, interquartile range. Statistical analysis was conducted using a Wilcoxon two-related samples test with a significance threshold set at *p* = 0.05. Sig., significance; NS, not significance. Hemoglobin, hematocrit, and triglyceride levels are reported, and all values fall within the normal physiological range for postmenopausal women.

### 3.4 Results of calcium concentration in serum, hair, and bone metabolism biomarkers

Table 4 presents the results of calcium concentration in serum, hair, and bone metabolism biomarkers of study participants before and after a 12-week intervention. Before the intervention, the placebo group exhibited significantly lower calcium concentration in serum compared to the probiotic group, although no significant differences were observed in calcium concentration in hair and bone metabolism biomarkers between the two groups at baseline.

**TABLE 4:** Calcium concentration in serum and hair and bone metabolism biomarkers from participating subjects at baseline and endline.

Variable	Group	Baseline		Endline		*P*-value	Sig.
		Mean ± SD	Median (IQR)	Mean ± SD	Median (IQR)		
Calcium in serum (mmol/L)	Placebo	1.90 ± 0.23	1.93 (1.87–2.02)	2.04 ± 0.09	2.03 (1.96–2.11)	0.00	Sig.
	Probiotic	2.10 ± 0.33	2.17 (2.05–2.26)	1.88 ± 0.44	1.98 (1.82–2.16)	0.03	Sig.
	*P*-value	0.02		0.64			
	Sig.	Sig.		NS			
Calcium in hairs (mg/g dry mass)	Placebo	2.52 ± 1.33	2.26 (1.32–3.70)	2.42 ± 1.50	2.44 (0.94–3.23)	0.43	NS
	Probiotic	2.42 ± 1.49	2.30 (1.27–3.42)	2.15 ± 1.36	2.21 (0.85–3.17)	0.19	NS
	*P*-value	0.76		0.45			
	Sig.	NS		NS			
BSAP (ng/ml)	Placebo	11.49 ± 2.91	11.16 (9.04–13.64)	11.38 ± 1.96	11.90 (9.62–12.69)	0.77	NS
	Probiotic	12.24 ± 2.92	12.19 (8.99–15.01)	10.90 ± 1.56	11.26 (9.04–12.33)	0.24	NS
	*P*-value	0.65		0.18			
	Sig.	NS		NS			
PINP (ng/ml)	Placebo	2.73 ± 0.45	2.58 (2.37–3.29)	2.66 ± 0.52	2.36 (2.26–3.18)	0.28	NS
	Probiotic	2.42 ± 0.12	2.39 (2.32–2.58)	2.64 ± 0.52	2.46 (2.39–2.59)	0.31	NS
	*P*-value	0.72		0.47			
	Sig.	NS		NS			
CTX (pg/ml)	Placebo	6.24 ± 0.94	6.05 (5.61–7.22)	5.11 ± 0.52	5.29 (4.70–5.49)	0.01	Sig.
	Probiotic	5.66 ± 0.15	5.63 (5.55–5.79)	5.67 ± 0.55	5.39 (5.31–5.87)	0.61	NS
	*P*-value	0.14		0.14			
	Sig.	NS		NS			
TRAP-5b (ng/ml)	Placebo	18.00 ± 6.93	17.36 (13.18–24.45)	21.13 ± 6.33	18.87 (15.80–27.32)	0.04	Sig.
	Probiotic	23.43 ± 9.19	21.26 (15.71–31.06)	26.09 ± 9.31	21.69 (18.12–35.82)	0.37	NS
	*P*-value	0.14		0.07			
	Sig.	NS		NS			

BSAP, bone-specific alkaline phosphatase; PINP, N-terminal propeptide of type I procollagen; CTX, C-terminal telopeptide of type I collagen; TRAP-5b, Tartrate-resistant acid phosphatase isoform-5b; SD, standard deviation; IQR, interquartile range. Statistical analysis was conducted using a Wilcoxon two-related samples test with a significance threshold set at *p* = 0.05. Sig., significance; NS, not significance.

At the endline period, no significant differences were found between the placebo and probiotic groups. Additionally, the placebo group exhibited a significant increase in serum calcium levels compared to the baseline period. Furthermore, after the 12-week intervention, the placebo group showed a significant decrease in CTX levels and a significant increase in TRAP-5b levels compared to the baseline period. In contrast, the probiotic group demonstrated a significant decrease in serum calcium levels compared to the baseline period. No significant differences were noted in hair calcium, bone resorption, and bone formation biomarkers within the probiotic group between the preintervention and postintervention periods.

### 3.5 Result of DXA assessment

Table 5 displays the BMD profiles of the lumbar spine, left femur, and total body before and after a 12-week intervention period. No significant changes in BMD levels were observed in either the placebo or probiotic groups from baseline to endline. However, it is worth noting that during the baseline period, the probiotic group exhibited a significantly lower BMD level at the left femur compared to the placebo group.

**TABLE 5:** Bone mineral density (BMD) profiles in dual-energy X-ray absorptiometry (DXA) assessment from participating subjects at baseline and endline.

Variable	Group	Baseline		Endline		*P*-value	Sig.
		Mean ± SD	Median (IQR)	Mean ± SD	Median (IQR)		
BMD Spine L1-L4 (g/cm^2^)	Placebo	1.20 ± 0.20	1.14 (1.01–1.38)	1.18 ± 0.21	1.11 (1.03–1.38)	0.35	NS
	Probiotic	1.14 ± 0.17	1.10 (0.99–1.26)	1.14 ± 0.17	1.08 (1.01–1.30)	0.20	NS
	*P*-value	0.22		0.30			
	Sig.	NS		NS			
BMD Left Femur (g/cm^2^)	Placebo	1.05 ± 0.13	1.03 (0.98–1.15)	1.05 ± 0.14	1.04 (0.97–1.14)	0.97	NS
	Probiotic	0.98 ± 0.12	0.97 (0.89–1.06)	0.98 ± 0.12	0.97 (0.88–1.05)	0.86	NS
	*P*-value	0.04		0.07			
	Sig.	Sig.		NS			
BMD Total body (g/cm^2^)	Placebo	1.16 ± 0.12	1.14 (1.04–1.25)	1.15 ± 0.11	1.17 (1.07–1.23)	0.67	NS
	Probiotic	1.13 ± 0.12	1.12 (1.02–1.22)	1.12 ± 0.12	1.12 (1.04–1.21)	0.24	NS
	*P*-value	0.40		0.39			
	Sig.	NS		NS			

L1–L4: the first to the fourth vertebra of the lumbar spine. BMD, bone mineral density, the amount of minerals measured by DXA. SD, standard deviation; IQR, interquartile range. Statistical analysis was conducted using a Wilcoxon two-related samples test with a significance threshold set at *p* = 0.05. Sig., significance; NS, not significance.

Table 6 presents a comparison of differences in BMD profiles assessed by DXA between the placebo and probiotic groups at baseline and endline. Although significant differences in the comparison between preintervention and postintervention periods were not observed in either the placebo or probiotic groups, it is noteworthy that the probiotic group, consumed by postmenopausal women for 12 weeks, demonstrated slightly larger differences in BMD across the spine, left femur, and total body, ranging from −0.01 to 0.00 g/cm^2^, compared to the placebo group, which showed no change.

**TABLE 6:** Comparison of differences in bone mineral density (BMD) profiles in DXA assessment between placebo and probiotic groups at baseline and endline.

Position	Variable	Placebo group	Probiotic group	*P*-value	Sig.
		Median (IQR)	Median (IQR)		
Spine L1-L4	Δ BMD (g/cm^2^)	0.00 (−0.03 to 0.01)	0.00 (−0.02 to 0.03)	0.33	NS
Left femur	Δ BMD (g/cm^2^)	0.00 (−0.02 to 0.01)	0.00 (−0.02 to 0.01)	0.67	NS
Total body	Δ BMD (g/cm^2^)	0.00 (−0.03 to 0.02)	−0.01 (−0.04 to 0.01)	0.76	NS

L1–L4: the first to the fourth vertebra of the lumbar spine. BMD, bone mineral density, the amount of minerals measured by DXA. Δ BMD, a difference value between before and after intervention. SD, standard deviation; IQR, interquartile range. Statistical analysis was conducted using a Wilcoxon two-related samples test with a significance threshold set at *p* = 0.05. Sig., significance; NS, not significance.

## 4 Discussion

Exploring dietary interventions may reveal new therapeutic targets for managing metabolic changes and bone health during menopause. It is important to note that although we hypothesized that probiotic supplementation with *L. acidophilus* would positively impact calcium status, bone metabolism biomarkers, and BMD profiles in postmenopausal women, our results did not fully support this hypothesis. Notably, we observed a concerning decrease in serum calcium levels in the probiotic group within this population. Our current study’s primary findings indicate that a 12-week daily probiotic *L. acidophilus* supplementation in postmenopausal women did not significantly influence the BMD profile. Additionally, our current findings highlighted that the observed values for various parameters, including hemoglobin, hematocrit, triglycerides, and glucose levels, remain within the normal physiological range for postmenopausal women. This suggests that while there may be some changes detected, major deviations from normal values are not to be expected.

Our main investigation extends to mineral status, with a particular focus on calcium and bone metabolism biomarkers. Menopause has been associated with changes in calcium balance and alterations in markers indicative of bone turnover (36). This study demonstrates a significant reduction in serum calcium concentrations among the probiotic group during the endline phase compared to the baseline phase, as depicted in Table 4. This result implies that consuming probiotic *L. acidophilus* may affect calcium balance in postmenopausal women. The observed decrease in serum calcium levels is consistent with findings from other studies, indicating the reliability of our results. Asemi and Esmaillzadeh (37), as well as Cheung et al. (38), have reported similar reductions in serum calcium and calcium absorption with probiotic interventions, suggesting a consistent trend in the impact of probiotics on calcium metabolism.

Moreover, in comparison to the placebo group, the probiotic group exhibited significantly increased energy, protein, and carbohydrate intake at the endline period, along with noticeable calcium intake before and after the intervention (Table 1). Surprisingly, despite significantly lower serum calcium levels in the probiotic group compared to the baseline period, this did not affect bone metabolism biomarkers (Table 3).

The mechanism underlying the decrease in serum calcium levels despite the high intake of carbohydrates, protein, phosphorus, and calcium involves several interconnected pathways. Firstly, phosphorus can form insoluble complexes with calcium in the intestines, thereby inhibiting calcium absorption. This reduced absorption contributes to lower circulating calcium levels. A reduced phosphorus intake triggers heightened synthesis of 1,25(OH)_2_D_3_, leading to enhanced phosphorus absorption in the intestines. Furthermore, the heightened levels of 1,25(OH)_2_D_3_ stimulate calcium absorption and raise serum calcium levels, subsequently reducing PTH levels and decreasing renal phosphorus excretion (39). Additionally, a high protein intake can elevate the excretion of calcium through the kidneys. This excessive calcium loss in urine diminishes the amount available in the bloodstream, further lowering serum calcium levels (40). Furthermore, increased protein intake can induce elevated acid production in the body, resulting in a state of metabolic acidosis (41, 42). These combined factors underscore the intricate balance among dietary composition, renal function, acid-base regulation, and bone metabolism in determining serum calcium levels.

In our previous study, we investigated the effects of probiotic *L. acidophilus* DSM079 supplementation in healthy female rats, which showed that this strain did not significantly impact serum calcium levels, calcium transport, and bone metabolism biomarker values (27). Although the exact mechanism behind the decrease in serum calcium levels due to probiotic supplementation was not directly addressed in the rat study, several factors could potentially contribute to this observation. One possible explanation could be the interaction between probiotics and gut microbiota composition, which might influence calcium absorption in the intestines. Probiotics have been demonstrated to alter nutrient absorption by influencing gut microbial ecology, and alterations in microbial populations could impact calcium uptake and metabolism. Additionally, probiotics might indirectly affect calcium homeostasis by influencing factors such as intestinal pH, nutrient transporters, or regulatory pathways involved in calcium absorption and utilization (43). Further investigation into these mechanisms is necessary to fully understand the relationship between probiotic supplementation and serum calcium levels in both animal models and human subjects.

In our current study, we observed significant fluctuations in bone resorption markers, particularly CTX and TRAP-5b, within the placebo group. These findings align with prior research, such as the study by Park et al. (44) which delineated the alterations of CTX levels across various menopausal stages. Their findings revealed an increase in CTX levels with progressing menopause duration, followed by a subsequent decrease in women experiencing menopause for over 10 years (44). Additionally, Gurban et al. (45) demonstrated a notable connection between menopausal duration and bone turnover markers, particularly TRAP-5b, in osteoporotic women (45). The intricate relationship between menopause and bone turnover markers involves hormonal shifts, notably the decline in estrogen levels, which diminishes the regulatory influence on osteoclast activity and bone resorption (46, 47). CTX, reflecting collagen degradation during bone resorption, and TRAP-5b, an enzyme released by osteoclasts, act as indicators of increased bone turnover and potential bone loss in postmenopausal women (48, 49). These studies highlight the significance of our findings and contribute to understanding the dynamics of bone metabolism in menopausal women.

This observation suggests that probiotic consumption might contribute to stabilizing the fluctuation of these bone turnover markers over the study duration, potentially offering benefits for maintaining bone health. Fluctuations in bone turnover markers, particularly significant increases in bone resorption markers or decreases in bone formation markers, often indicate adverse effects on bone density and integrity, increasing the risk of conditions such as osteoporosis (50). By observing these fluctuations, it is suggested that consuming probiotics like *L. acidophilus* for 3 months may help mitigate the negative impacts of menopause, as evidenced by the higher concentrations of bone resorption in the placebo group.

The underlying mechanisms responsible for the observed stabilization of bone turnover markers by probiotic *L. acidophilus* are still not fully understood. Previous studies have reported potential mechanisms through which *L. acidophilus* could impact markers of bone turnover. These mechanisms often involve the modulation of gut microbiota (51–54), enhancement of mineral absorption (55), and production of bioactive compounds (56, 57) that affect bone health. The specific mechanisms may vary depending on factors such as the composition of the gut microbiota (58), individual differences (59), and the duration of intervention (60). It is worth noting that the 12-week duration of our intervention may be relatively short. In addition, our study cohort consisted of postmenopausal women without diagnosed osteoporosis, which could have influenced the observed outcomes.

In another finding from our current investigation, the consumption of probiotic *L. acidophilus* for 12 weeks did not have a significant impact on BMD levels at the lumbar spine, left femur, and total body between the preintervention and postintervention periods (Table 5). Despite demonstrating significant calcium intake before and after the intervention (Table 1), these variations in nutrient intake did not seem to affect BMD in postmenopausal women. However, it is worth noting that significant changes were observed in body fat composition. Typically, the loss of body fat is linked to improved bone health (61). However, the relatively short duration of 12 weeks in our study may have limited our ability to confirm such an association.

Our current study revealed a significant reduction in the percentage of body fat and visceral fat in the probiotic group between preintervention and postintervention assessments, as shown in Table 2. Insights from Kang et al. (62) shed light on the potential impact of *L. acidophilus* on body weight and fat mass. In their study, mice fed a high-fat diet experienced a decrease in body weight and fat mass with *L. acidophilus* consumption. This effect coincided with the activation of brown adipose tissue, suggesting a role for *L. acidophilus* in modulating adiposity and potentially influencing metabolic processes. This reduction underscores the potential impact of probiotic *L. acidophilus* consumption on body composition and distribution of adipose tissue in postmenopausal women, suggesting avenues for future research.

Additionally, it is essential to consider the potential link between the observed changes in body composition and glucose levels. Typically, reductions in fat mass are associated with lower glucose levels (63). However, our current study revealed a significant increase in blood glucose concentration within the probiotic group from preintervention to postintervention, as demonstrated in Table 3. In addition, the probiotic group exhibited significantly higher energy, protein, and carbohydrate intakes compared to the placebo group (Table 1). Increased dietary energy and carbohydrate intake can raise blood glucose levels by stimulating insulin secretion and promoting glucose production through glycogenolysis and gluconeogenesis (64). Similarly, higher protein intake may contribute to elevated blood glucose through the gluconeogenic pathway, as amino acids can be converted into glucose in the liver (65). The findings of our current study align with previous research, indicating similar effects of probiotic consumption on glucose metabolism. For instance, the inclusion of probiotic yogurt containing *L. acidophilus* La5 and *B. animalis* subsp *lactis* Bb12 led to a significantly higher fasting glucose level in overweight men and women (66). These outcomes are consistent with a meta-analysis reviewing the effects of oral probiotic supplementation in postmenopausal women, which reported a nonsignificant reduction in glucose (67).

The observed increase in glucose levels in the probiotic group could potentially stem from several factors. Firstly, probiotics might affect glucose metabolism through interactions with the gut microbiota. Alterations in gut microbiota composition can influence carbohydrate fermentation and the production of short-chain fatty acids, which play a role in glucose homeostasis (68). Additionally, the decrease in serum calcium levels observed in the probiotic group may indirectly impact glucose metabolism. Calcium is involved in insulin secretion and sensitivity, and alterations in calcium levels can affect glucose uptake and utilization by pancreatic β-cells (69). Therefore, the decrease in serum calcium levels might have contributed to the observed increase in glucose concentration. Moreover, the slightly increased HOMA-IR index in the probiotic group suggests alterations in insulin resistance, which could further contribute to higher glucose levels. Overall, these findings suggest a complex interplay between probiotic supplementation, calcium metabolism, and glucose homeostasis, underscoring the need for further research to elucidate the underlying mechanisms. Variations in factors such as the type of probiotic administered (e.g., yogurt or capsules), the duration of the intervention, and the diversity of probiotic strains utilized could help clarify the disparities observed in research findings (67). Nevertheless, it is essential to acknowledge that participants in our study might have adjusted their dietary habits, a common occurrence during consultations with healthcare professionals. We did not monitor their dietary intake throughout the entire 12-week period. While the exact degree of importance attributed to these adjustments remains unclear, future research should aim to explore their potential impact on the outcomes of interest in similar studies. This acknowledgment underscores the need for further investigation into the relationship between dietary adjustments and study outcomes, providing valuable insights for the interpretation and generalization of study findings.

### 4.1 Study limitations and future perspectives

The strengths of our study are rooted in its rigorous methodology, which includes a controlled and randomized clinical design, meticulous measurement of key parameters, and comprehensive analysis of outcomes. Additionally, the use of advanced devices like the InBody for body composition assessment and DXA as the gold standard for assessing BMD enhances the reliability and precision of our findings. These robust methodological approaches bolster the reliability and validity of our findings, providing valuable insights into the potential clinical implications of probiotic supplementation in this demographic.

While our study represents a significant step forward, it is crucial to acknowledge certain limitations. These include the omission of measurements for inflammatory cytokine markers (70) and short-chain fatty acids (56), which are essential for a comprehensive understanding of the gut microbiota–bone interaction. Additionally, a detailed exploration of gut microbiota composition (51), intervention duration (59), and calcium transporter markers (71) was beyond the scope of our study, allowing for further investigation in subsequent research endeavors.

Furthermore, the short-term intervention period of 3 months in our study might not entirely capture the long-term benefits of probiotic consumption for menopausal women. Prior studies have indicated the beneficial effects of probiotics on bone health following longer intervention durations, spanning from 6 months (21, 70) to 12 months (72–74). Future studies with extended intervention periods could offer valuable insights into the sustained impact of probiotic supplementation on bone health outcomes in postmenopausal women.

Therefore, while our study provides valuable insights into the effects of probiotic supplementation on calcium status, bone metabolism biomarkers, and bone mineral density profiles in postmenopausal women, several limitations must be addressed. Firstly, we acknowledge that not screening for osteoporosis at baseline could impact the interpretation of our results, given that menopause is a known risk factor for osteoporosis. Secondly, although randomization was employed to minimize confounders, the lack of homogeneity in the study group remains a concern, potentially introducing variability among participants. Additionally, we recognize the importance of analyzing the composition of the gut microbiome at baseline and post-intervention to assess the effects of administering *L. acidophilus*, a consideration for future research. Furthermore, while our study was conducted over a 12-week period, future studies with longer durations at least a year are warranted to evaluate the sustained benefits of probiotic administration on overall bone health. Lastly, self-reported data may be prone to recall bias and might not accurately capture the intricacies of participants’ dietary habits. Moreover, relying solely on questionnaires lacks real-time or objective measures of dietary intake, potentially introducing inaccuracies into the analysis. Additionally, our study did not include continuous monitoring of dietary intake throughout the entire 12-week intervention period, representing another limitation. Continuous monitoring could offer valuable insights into changes in dietary patterns over time and their impact on study outcomes. By delving into these unexplored facets, researchers can contribute to the development of more effective nutritional interventions aimed at improving bone health in postmenopausal women.

Our study carries significant clinical implications for the bone health of postmenopausal women. The findings highlight the potential advantages of probiotic supplementation in managing bone health parameters. Understanding the effects of probiotics on calcium status, bone metabolism biomarkers, and BMD profiles offers valuable insights for healthcare providers. Integrating probiotic supplementation into tailored nutritional interventions for postmenopausal women could potentially reduce the risk of osteoporotic fractures and improve overall bone health. This study adds to the increasing body of evidence advocating for the incorporation of probiotics into clinical practice to optimize bone health outcomes in this population.

## 5 Conclusion

In conclusion, daily consumption of oral probiotic *L. acidophilus* UALa-01™ for 12 weeks in postmenopausal women does not affect the BMD profile, but it may prevent fluctuations in bone turnover. However, such supplementation can disturb calcium and glucose status in postmenopausal women.

Further research is necessary to explore the long-term implications of probiotic consumption on calcium metabolism for postmenopausal women, to fully understand the potential benefits of probiotic interventions in this population.

## Data availability statement

The raw data supporting the conclusions of this article will be made available by the authors, without undue reservation.

## Ethics statement

The studies involving humans were approved by the Ethics Committee of Poznań University of Medical Sciences—Poland (approval no. 668/21 issued on 23 September 2021). The studies were conducted in accordance with the local legislation and institutional requirements. Written informed consent for participation in this study was provided by the participants’ legal guardians/next of kin. Written informed consent was obtained from the individual(s) for the publication of any potentially identifiable images or data included in this article.

## Author contribution

IH: Conceptualization, Data curation, Formal analysis, Funding acquisition, Investigation, Methodology, Project administration, Resources, Validation, Visualization, Writing – original draft, Writing – review & editing. MM: Formal analysis, Investigation, Methodology, Writing – review & editing. MC-M: Formal analysis, Investigation, Methodology, Writing – review & editing. KS: Investigation, Methodology, Project administration, Writing – review & editing. PB: Methodology, Supervision, Writing – review & editing. JS: Conceptualization, Investigation, Methodology, Supervision, Validation, Writing – review & editing.

## Footnotes

1
https://clinicaltrials.gov/ct2/show/NCT05332626

## References

[1] Daan N Fauser B. Menopause prediction and potential implications. *Maturitas.* (2015) 82:257–65. 10.1016/j.maturitas.2015.07.019 26278873

[2] Akkawi I Zmerly H. Osteoporosis: Current concepts. *Joints.* (2018) 06:122–7. 10.1055/s-0038-1660790 30051110 PMC6059859

[3] Aspray T Hill T. Osteoporosis and the ageing skeleton. *Subcell Biochem.* (2019) 91:453–76. 10.1007/978-981-13-3681-2_16 30888662

[4] Kanis J Kanis J. Assessment of fracture risk and its application to screening for postmenopausal osteoporosis: Synopsis of a WHO report. *Osteoporos Int.* (1994) 4:368–81. 10.1007/BF01622200 7696835

[5] Rao A Reddy S Rao D. Is there a difference between right and left femoral bone density? *J Clin Densitom.* (2000) 3:57–61. 10.1385/JCD:3:1:057 10745302

[6] Gkastaris K Goulis D Potoupnis M Anastasilakis A Kapetanos G. Obesity, osteoporosis and bone metabolism. *J Musculoskelet Neuronal Interact.* (2020) 20:372–81. 32877973 PMC7493444

[7] Lorentzon M Johansson H Harvey N Liu E Vandenput L McCloskey EV Osteoporosis and fractures in women: The burden of disease. *Climacteric.* (2022) 25:4–10. 10.1080/13697137.2021.1951206 34319208

[8] Mahdavi-Roshan M Ebrahimi M Ebrahimi A. Copper, magnesium, zinc and calcium status in osteopenic and osteoporotic post-menopausal women. *Clin Cases Miner Bone Metab.* (2015) 12:18–21. 10.11138/ccmbm/2015.12.1.018 26136790 PMC4469220

[9] Song L. *Calcium and bone metabolism indices.* 1st ed. Amsterdam: Elsevier Inc (2017). p. 1–46. 10.1016/bs.acc.2017.06.005 28939209

[10] Wasilewski G Vervloet M Schurgers L. The bone–vasculature axis: Calcium supplementation and the role of vitamin K. *Front Cardiovasc Med.* (2019) 6:1–16. 10.3389/fcvm.2019.00006 30805347 PMC6370658

[11] Park S Kang S Kim D. Severe calcium deficiency increased visceral fat accumulation, down-regulating genes associated with fat oxidation, and increased insulin resistance while elevating serum parathyroid hormone in estrogen-deficient rats. *Nutr Res.* (2020) 73:48–57. 10.1016/j.nutres.2019.09.008 31841747

[12] Sood R Faubion S Kuhle C Thielen J Shuster L. Prescribing menopausal hormone therapy: An evidence-based approach. *Int J Womens Health.* (2014) 6:47–57. 10.2147/IJWH.S38342 24474847 PMC3897322

[13] Reyes C Hitz M Prieto-Alhambra D Abrahamsen B. Risks and benefits of bisphosphonate therapies. *J Cell Biochem.* (2016) 117:20–8. 10.1002/jcb.25266 26096687

[14] Pinkerton JV Thomas S. Use of SERMs for treatment in postmenopausal women. *J Steroid Biochem Mol Biol.* (2014) 142:142–54. 10.1016/j.jsbmb.2013.12.011 24373794

[15] Vieira A Castelo P Ribeiro D Ferreira C. Influence of oral and gut microbiota in the health of menopausal women. *Front Microbiol.* (2017) 8:1884. 10.3389/fmicb.2017.01884 29033921 PMC5625026

[16] He Y Chen Y. The potential mechanism of the microbiota-gut-bone axis in osteoporosis: A review. *Osteoporos Int.* (2022) 33:2495–506. 10.1007/s00198-022-06557-x 36169678

[17] Harahap I Suliburska J. Probiotics and isoflavones as a promising therapeutic for calcium status and bone health: A narrative review. *Foods.* (2021) 10:2685. 10.3390/foods10112685 34828966 PMC8621960

[18] Laird E Molloy A McNulty H Ward M McCarroll K Hoey L Greater yogurt consumption is associated with increased bone mineral density and physical function in older adults. *Osteoporos Int.* (2017) 28:2409–19. 10.1007/s00198-017-4049-5 28462469

[19] Zhao F Guo Z Kwok L Zhao Z Wang K Li Y *Bifidobacterium lactis* Probio-M8 improves bone metabolism in patients with postmenopausal osteoporosis, possibly by modulating the gut microbiota. *Eur J Nutr.* (2023) 62:965–76. 10.1007/s00394-022-03042-3 36334119

[20] Harahap I Suliburska J. Can probiotics decrease the risk of postmenopausal osteoporosis in women? *PharmaNutrition.* (2023) 24:100336. 10.1016/j.phanu.2023.100336

[21] Jafarnejad S Djafarian K Fazeli M Yekaninejad M Rostamian A Keshavarz S. Effects of a multispecies probiotic supplement on bone health in osteopenic postmenopausal women: A randomized, double-blind, controlled trial. *J Am Coll Nutr.* (2017) 36:497–506. 10.1080/07315724.2017.1318724 28628374

[22] Gholami A Dabbaghmanesh M Ghasemi Y Koohpeyma F Talezadeh P Montazeri-Najafabady N. The ameliorative role of specific probiotic combinations on bone loss in the ovariectomized rat model. *BMC Complement Med Ther.* (2022) 22:1–11. 10.1186/s12906-022-03713-y 36115982 PMC9482298

[23] Malmir H Ejtahed H Soroush A Mortazavian A Fahimfar N Ostovar A Probiotics as a new regulator for bone health: A systematic review and meta-analysis. *Evid Based Complement Altern Med.* (2021) 2021:2989. 10.1155/2021/3582989 34394379 PMC8355998

[24] Dar H Shukla P Mishra P Anupam R Mondal R Tomar G *Lactobacillus acidophilus* inhibits bone loss and increases bone heterogeneity in osteoporotic mice via modulating Treg-Th17 cell balance. *Bone Rep.* (2018) 8:46–56. 10.1016/j.bonr.2018.02.001 29955622 PMC6019967

[25] Harahap I Olejnik A Kowalska K Suliburska J. Effects of daidzein, tempeh, and a probiotic digested in an artificial gastrointestinal tract on calcium deposition in human osteoblast-like saos-2 cells. *Int J Mol Sci.* (2024) 25:1008. 10.3390/ijms25021008 38256081 PMC10815870

[26] Harahap I Kuligowski M Schmidt M Kurzawa P Suliburska J. Influence of isoflavones and probiotics on magnesium status in healthy female rats. *Foods.* (2023) 12:3908. 10.3390/foods12213908 37959026 PMC10647356

[27] Harahap I Kuligowski M Schmidt M Kołodziejski P Suliburska J. Effects of isoflavone and probiotic intake on calcium transport and bone metabolism biomarkers in female rats. *Food Sci Nutr.* (2023) 11:6324–35. 10.1002/fsn3.3571 37823105 PMC10563734

[28] Harahap I Kuligowski M Schmidt M Suliburska J. The impact of soy products, isoflavones, and *Lactobacillus acidophilus* on iron status and morphological parameters in healthy female rats. *J Trace Elem Med Biol.* (2023) 78:127183. 10.1016/j.jtemb.2023.127183 37120971

[29] Harahap I Kuligowski M Schmidt M Suliburska J. The impact of soybean products and probiotics on calcium bioaccessibility from organic and inorganic calcium salts in an in vitro digestion model. *Food Chem Adv.* (2023) 2:100269. 10.1016/j.focha.2023.100269

[30] Harahap I Kuligowski M Schmidt M Brzozowska A Suliburska J. Impact of isoflavones and *Lactobacillus acidophilus* on the fecal microbiology status in healthy female rats. *Acta Sci Pol Technol Aliment.* (2022) 21:223–31. 10.17306/J.AFS.2022.1059

[31] Harahap I Kuligowski M Schmidt M Kurzawa P Pruszyńska-Oszmałek E Sassek M Isoflavones and probiotics effect on bone calcium and bone cells in rats. *Heliyon.* (2023) 9:e16801. 10.1016/j.heliyon.2023.e16801 37292353 PMC10245251

[32] Pirayesh Islamian J Garoosi I Abdollahi Fard K Abdollahi M. Comparison between the MDCT and the DXA scanners in the evaluation of BMD in the lumbar spine densitometry. *Egypt J Radiol Nucl Med.* (2016) 47:961–7. 10.1016/j.ejrnm.2016.04.005

[33] Kang H Im S. Probiotics as an immune modulator. *J Nutr Sci Vitaminol.* (2015) 61:S103–5. 10.3177/jnsv.61.S103 26598815

[34] Andreoli A Garaci F Cafarelli F Guglielmi G. Body composition in clinical practice. *Eur J Radiol.* (2016) 85:1461–8. 10.1016/j.ejrad.2016.02.005 26971404

[35] Soleimani A Zarrati Mojarrad M Bahmani F Taghizadeh M Ramezani M Tajabadi-Ebrahimi M Probiotic supplementation in diabetic hemodialysis patients has beneficial metabolic effects. *Kidney Int.* (2017) 91:435–42. 10.1016/j.kint.2016.09.040 27927601

[36] Honour J. Biochemistry of the menopause. *Ann Clin Biochem.* (2018) 55:18–33. 10.1177/0004563217739930 29027807

[37] Asemi Z Esmaillzadeh A. Effect of daily consumption of probiotic yoghurt on serum levels of calcium, Iron and liver enzymes in pregnant women. *Int J Prev Med.* (2013) 4:949–55. 24049622 PMC3775173

[38] Cheung A Wilcox G Walker K Shah N Strauss B Ashton J Fermentation of calcium-fortified soya milk does not appear to enhance acute calcium absorption in osteopenic post-menopausal women. *Br J Nutr.* (2011) 105:282–6. 10.1017/S0007114510003442 20854699

[39] Taylor J Bushinsky D. Calcium and phosphorus homeostasis. *Blood Purif.* (2009) 27:387–94. 10.1159/000209740 19299893

[40] Martin W Armstrong L Rodriguez N. Dietary protein intake and renal function. *Nutr Metab.* (2005) 2:25. 10.1186/1743-7075-2-25 16174292 PMC1262767

[41] Williams R Kozan P Samocha-Bonet D. The role of dietary acid load and mild metabolic acidosis in insulin resistance in humans. *Biochimie.* (2016) 124:171–7. 10.1016/j.biochi.2015.09.012 26363101

[42] Reddy S Wang C Sakhaee K Brinkley L Pak C. Effect of low-carbohydrate high-protein diets on acid-base balance, stone-forming propensity, and calcium metabolism. *Am J Kidney Dis.* (2002) 40:265–74. 10.1053/ajkd.2002.34504 12148098

[43] Wang J Wu S Zhang Y Yang J Hu Z. Gut microbiota and calcium balance. *Front Microbiol.* (2022) 13:1033933. 10.3389/fmicb.2022.1033933 36713159 PMC9881461

[44] Park S Jeong S Lee J Ryu S Jeong H Sim Y The changes of CTX, DPD, osteocalcin, and bone mineral density during the postmenopausal period. *Ann Rehabil Med.* (2018) 42:441–8. 10.5535/arm.2018.42.3.441 29961742 PMC6058582

[45] Gurban C Balaş M Vlad M Caraba A Jianu A Bernad E Bone turnover markers in postmenopausal osteoporosis and their correlation with bone mineral density and menopause duration. *Rom J Morphol Embryol.* (2019) 60:1127–35. 32239087

[46] Khosla S. Estrogen versus FSH effects on bone metabolism: Evidence from interventional human studies. *Endocrinology.* (2020) 161:1–9. 10.1210/endocr/bqaa111 32602895 PMC7371389

[47] Mathis K Sturgeon K Winkels R Wiskemann J De Souza M Schmitz K. Bone resorption and bone metastasis risk. *Med Hypotheses.* (2018) 118:36–41. 10.1016/j.mehy.2018.06.013 30037612

[48] Löfvall H Newbould H Karsdal M Dziegiel M Richter J Henriksen K Osteoclasts degrade bone and cartilage knee joint compartments through different resorption processes. *Arthritis Res Ther.* (2018) 20:67. 10.1186/s13075-018-1564-50 PMC5894194 29636095

[49] Lipton A Chapman J Demers L Shepherd L Han L Wilson C Elevated bone turnover predicts for bone metastasis in postmenopausal breast cancer: Results of NCIC CTG MA.14. *J Clin Oncol.* (2011) 29:3605–10. 10.1200/JCO.2010.31.5069 21859992

[50] Wheater G Elshahaly M Tuck S Datta H van Laar J. The clinical utility of bone marker measurements in osteoporosis. *J Transl Med.* (2013) 11:201. 10.1186/1479-5876-11-201 23984630 PMC3765909

[51] He M Shi B. Gut microbiota as a potential target of metabolic syndrome: The role of probiotics and prebiotics. *Cell Biosci.* (2017) 7:1–14. 10.1186/s13578-017-0183-1 29090088 PMC5655955

[52] Brahe L Le Chatelier E Prifti E Pons N Kennedy S Blædel T Dietary modulation of the gut microbiota - A randomised controlled trial in obese postmenopausal women. *Br J Nutr.* (2015) 114:406–17. 10.1017/S0007114515001786 26134388 PMC4531470

[53] Azad M Sarker M Li T Yin J. Probiotic species in the modulation of gut microbiota: An overview. *Biomed Res Int.* (2018) 2018:9478630. 10.1155/2018/9478630 29854813 PMC5964481

[54] Chen J Liu X Li S Li J Fang G Chen Y Effects of *Lactobacillus acidophilus* and *L. reuteri* on bone mass and gut microbiota in ovariectomized mice. *Cell Mol Biol.* (2023) 69:43–51. 10.14715/cmb/2023.69.9.7 37807335

[55] Varvara R Vodnar D. Probiotic-driven advancement: Exploring the intricacies of mineral absorption in the human body. *Food Chem X.* (2024) 21:101067. 10.1016/j.fochx.2023.101067 38187950 PMC10767166

[56] Cheng Y Liu J Ling Z. Short-chain fatty acids-producing probiotics: A novel source of psychobiotics. *Crit Rev Food Sci Nutr.* (2022) 62:7929–59. 10.1080/10408398.2021.1920884 33955288

[57] Markowiak-Kope P Slizewska K. The trend in the amount of SCFAs found in feces is more closely related to nutrition, environmental variables, and intestinal microbiome dysbiosis. *Nutrients.* (2020) 12:1–23.

[58] O’Connor E O’Herlihy E O’Toole P. Gut microbiota in older subjects: Variation, health consequences and dietary intervention prospects. *Proc Nutr Soc.* (2014) 73:441–51. 10.1017/S0029665114000597 24824449

[59] Lampe J Navarro S Hullar M Shojaie A. Inter-individual differences in response to dietary intervention: Integrating omics platforms towards personalised dietary recommendations. *Proc Nutr Soc.* (2013) 72:207–18. 10.1017/S0029665113000025 23388096 PMC3694579

[60] Xu Z Knight R. Dietary effects on human gut microbiome diversity. *Br J Nutr.* (2015) 113:S1–5. 10.1017/S0007114514004127 25498959 PMC4405705

[61] Holick M Nieves J. Nutrition and bone health. *Nutr Bone Heal.* (2015) 12u78:1–7. 10.1007/978-1-4939-2001-3

[62] Kang Y Kang X Yang H Liu H Yang X Liu Q *Lactobacillus acidophilus* ameliorates obesity in mice through modulation of gut microbiota dysbiosis and intestinal permeability. *Pharmacol Res.* (2022) 175:106020. 10.1016/j.phrs.2021.106020 34896249

[63] Gower B Hunter G Chandler-Laney PC Alvarez JA Bush NC. Glucose metabolism and diet predict changes in adiposity and fat distribution in weight-reduced women. *Obesity.* (2010) 18:1532–7. 10.1038/oby.2009.459 20035282 PMC3070365

[64] Russell W Baka A Björck I Delzenne N Gao D Griffiths H Impact of diet composition on blood glucose regulation. *Crit Rev Food Sci Nutr.* (2016) 56:541–90. 10.1080/10408398.2013.792772 24219323

[65] Arrieta-Cruz I Gutiérrez-Juárez R. The role of circulating amino acids in the hypothalamic regulation of liver glucose metabolism. *Adv Nutr.* (2016) 7:790S–7S. 10.3945/an.115.011171 27422516 PMC4942863

[66] Ivey K Hodgson J Kerr D Lewis J Thompson P Prince R. The effects of probiotic bacteria on glycaemic control in overweight men and women: A randomised controlled trial. *Eur J Clin Nutr.* (2014) 68:447–52. 10.1038/ejcn.2013.294 24569536

[67] Li Z Li Y Pan B Wang X Wu Y Guo K The effects of oral probiotic supplementation in postmenopausal women with overweight and obesity: A systematic review and meta-analysis of randomized controlled trials. *Probiot Antimicrob Proteins.* (2023) 15:1567–82. 10.1007/s12602-022-10037-3 36576686

[68] Falcinelli S Rodiles A Hatef A Picchietti S Cossignani L Merrifield D Influence of probiotics administration on gut microbiota core. *J Clin Gastroenterol.* (2018) 52:S50–6. 10.1097/MCG.0000000000001064 29864068

[69] Gilon P Chae H Rutter G Ravier M. Calcium signaling in pancreatic β-cells in health and in type 2 diabetes. *Cell Calcium.* (2014) 56:340–61. 10.1016/j.ceca.2014.09.001 25239387

[70] Takimoto T Hatanaka M Hoshino T Takara T Tanaka K Shimizu A Effect of *Bacillus subtilis* C-3102 on bone mineral density in healthy postmenopausal Japanese women: A randomized, placebo-controlled, double-blind clinical trial. *Biosci Microbiota Food Heal.* (2018) 37:87–96. 10.12938/bmfh.18-006 PMC6200670 30370192

[71] Raveschot C Coutte F Frémont M Vaeremans M Dugersuren J Demberel S Probiotic *Lactobacillus* strains from Mongolia improve calcium transport and uptake by intestinal cells in vitro. *Food Res Int.* (2020) 133:109201. 10.1016/j.foodres.2020.109201 32466902

[72] Han H Kim J Choi Y Lee K Kwon T Kim S. Effect of *Lactobacillus fermentum* as a probiotic agent on bone health in postmenopausal women. *J Bone Metab.* (2022) 29:225–33. 10.11005/jbm.2022.29.4.225 36529865 PMC9760773

[73] Jansson P Curiac D Lazou Ahrén I Hansson F Martinsson Niskanen T Sjögren K Probiotic treatment using a mix of three *Lactobacillus* strains for lumbar spine bone loss in postmenopausal women: A randomised, double-blind, placebo-controlled, multicentre trial. *Lancet Rheumatol.* (2019) 1:e154–62. 10.1016/S2665-9913(19)30068-2 38229392

[74] Li P Sundh D Ji B Lappa D Ye L Nielsen J Metabolic alterations in older women with low bone mineral density supplemented with *Lactobacillus reuteri*. *JBMR Plus.* (2021) 5:1–14. 10.1002/jbm4.10478 33869994 PMC8046097

